# Immunometabolic Dysregulation in Female Obesity: Mechanistic Links to Ovarian Dysfunction, Implantation Failure, and Preeclampsia

**DOI:** 10.3390/biomedicines14092039

**Published:** 2026-09-10

**Authors:** Rodolfo Oliveira Medeiros, Feres Abrão, Laila Abrão, Cintia Gisele de Andrade Pozenato, Camila Abrão Costa Buzeto, Marines Laveso de Brito, Isis Carrero Zequini de Freitas, Vitória Auler do Santo, Gustavo Henrique de Paulo Ribeiro Ponciano, Bianca Marques, Juliana Ferreira Marcandelli, Isabele de Assis Nagahashi Campos, Maria Clara de Castro Ferreira, Gabriela Novaes Garcia, Cynthia de Paula Costa Borba, José Antonio Pizzolato Neto, Ludmila Trambaiolli de Souza, Felipe Ravazzi Guzzo, Amanda Santiago Ribeiro, Pedro Henrique Lima Domingues, Kelly Karine Pasqual, Felipe Neves Brandão

**Affiliations:** 1 Undergraduate Medical Program, Universidade de Marília (UNIMAR), Marília 17525-902, SP, Brazil; feresabrao@terra.com.br (F.A.); lailabrao3@gmail.com (L.A.); cintia.gisele@hotmail.com (C.G.d.A.P.); marineslaveso15@gmail.com (M.L.d.B.); vitoriaaulerdosanto@unimar.br (V.A.d.S.); gustavoponciano@gmail.com (G.H.d.P.R.P.); biancamarques.2503@gmail.com (B.M.); marcandellijuliana@gmail.com (J.F.M.); isabelle.nagahashi@icloud.com (I.d.A.N.C.); maclacastrosantos@hotmail.com (M.C.d.C.F.); gabrielapirajagarcia@gmail.com (G.N.G.); cy.mp@hotmail.com (C.d.P.C.B.); netopizzolato@gmail.com (J.A.P.N.); trambaiolliludmila@gmail.com (L.T.d.S.); feliperavazzi2020@gmail.com (F.R.G.); amandinhax12@hotmail.com (A.S.R.); pedrin_lima@hotmail.com (P.H.L.D.); kellypasqual@gmail.com (K.K.P.); fenebrandao@gmail.com (F.N.B.); 2 Undergraduate Medical Program, Faculdade de Medicina de Marília (FAMEMA), Marília 17519-030, SP, Brazil; fbuzeto1@gmail.com (C.A.C.B.); isiszequini@hotmail.com (I.C.Z.d.F.)

**Keywords:** female obesity, chronic low-grade inflammation, insulin resistance, hyperandrogenemia, reproductive immunology, ovarian dysfunction, endometrial receptivity, preeclampsia, infertility

## Abstract

Obesity is one of the most pressing public health challenges of our time, with a growing impact on metabolic and reproductive dysfunction in women. The expansion of visceral adipose tissue drives a chronic, low-grade systemic inflammatory state that has been associated with functional changes in the ovary, the endometrium, and the placenta across different stages of the reproductive process. This structured narrative review, guided by a PICo-framed research question and literature searches across PubMed/MEDLINE, Scopus, Web of Science, Embase, and ScienceDirect, examined the main immunometabolic mechanisms underlying female obesity and their reported associations with ovarian dysfunction, implantation failure, placental dysfunction, infertility, and preeclampsia. The evidence indicates that chronic low-grade inflammation, insulin resistance (IR), and functional hyperandrogenemia may impair folliculogenesis, oocyte quality, and ovarian reserve, while cytokine and adhesion-molecule dysregulation in the endometrium has been linked to impaired decidualization and a narrower implantation window. At the maternal–fetal interface, this same inflammatory substrate has been associated with deficient trophoblast invasion, inadequate spiral artery remodeling, and angiogenic imbalance, mechanisms widely implicated in the pathophysiology of preeclampsia. Rather than demonstrating a proven longitudinal chain within the same individual, the evidence reviewed here, drawn from heterogeneous populations, study designs, and experimental models, supports a conceptual, hypothesis-generating model in which obesity-related systemic inflammation may contribute to dysfunction across the ovary, endometrium, and placenta. Clinically, these findings support further investigation of reproductive biomarkers and early screening strategies for women with obesity, particularly in the context of assisted reproduction, alongside emerging preconception therapeutic approaches that remain largely investigational. Female obesity should therefore be understood not merely as a metabolic disorder, but as an immunometabolic condition potentially associated with dysfunction across the ovary–endometrium–placenta axis, an integrative framework that may help guide future preventive and therapeutic strategies for reproductive health, pending confirmation in longitudinal studies.

## 1. Introduction

Obesity is one of the most significant public health challenges of our time, with a growing impact on global morbidity and mortality, largely because of its strong ties to metabolic, cardiovascular, and reproductive disease. Although obesity has traditionally been studied from a general metabolic standpoint, emerging evidence points to sex-specific consequences for reproductive physiology in women, consequences that remain underappreciated as drivers of infertility and pregnancy complications [1,2].

In women, the expansion of visceral adipose tissue creates a pro-inflammatory systemic environment whose effects have been associated with functional changes in the ovary, the endometrium, and the maternal–placental unit, although the available evidence does not establish a single, obligatory pathway linking these sites within the same individual. This pattern of metabolic dysfunction interacts with hormonal changes, including functional hyperandrogenemia and insulin resistance (IR), and has been associated with impaired folliculogenesis, oocyte quality, and endometrial receptivity, factors implicated in the multifactorial infertility seen in obesity [2,3]. Once pregnancy is established, this same inflammatory substrate has been linked to deficient trophoblast invasion, inadequate spiral artery remodeling, and an imbalance between pro- and anti-angiogenic factors, which may favor inadequate placentation and an exaggerated systemic vascular response. This supports the view that maternal obesity should be regarded as a chronic inflammatory condition that may carry obstetric consequences [4,5].

Given this, a comprehensive and integrative understanding of the immunometabolic mechanisms linked to female obesity is essential, from their systemic origin to their reported associations with ovarian, endometrial, and placental function. This review therefore set out to examine the main immunometabolic mechanisms involved in female obesity, with emphasis on their reported associations with ovarian dysfunction, implantation failure, placental dysfunction, infertility, and preeclampsia, while highlighting clinical implications and emerging, largely investigational, therapeutic directions for reproductive health.

## 2. Search Strategy

This is a structured narrative review, carried out according to methodological principles used in evidence-based reviews, with the aim of supporting consistency and analytical depth in synthesizing the scientific evidence. It was not designed or conducted as a systematic review, and no formal risk-of-bias assessment, dual independent screening, or PRISMA-type reporting was performed [6].

The guiding research question was built around an adapted PICo framework: “What are the main immunometabolic mechanisms associated with female obesity, and how do these mechanisms impair ovarian function, endometrial receptivity, and placental function, thereby influencing infertility and the development of preeclampsia?” Within this framework, the population (P) was women with obesity of reproductive age, the phenomenon of interest (I) was immunometabolic mechanisms with reproductive consequences, and the context (Co) was the development of adverse reproductive and obstetric outcomes [7]. The PICo framework, rather than the conventional PICO (Population, Intervention, Comparison, Outcome) framework typically used for intervention-focused questions, was selected because this narrative review aimed to explore and integrate mechanistic and clinical evidence on obesity-related immunometabolic dysfunction across reproductive contexts, rather than to evaluate the comparative effectiveness of a predefined intervention against a comparator with pre-specified outcomes.

The literature search was carried out across the major biomedical databases: PubMed/MEDLINE, Scopus, and Web of Science, complemented by ScienceDirect and Embase to increase sensitivity and reduce selection bias. Controlled descriptors and keywords were combined through Boolean operators, including the terms “female obesity,” “visceral adiposity,” “ovarian dysfunction,” “endometrial receptivity,” “implantation failure,” “placental dysfunction,” “preeclampsia,” and “infertility,” with the search restricted to studies published between 2015 and 2026 in view of how rapidly knowledge of reproductive immunometabolism has advanced, prioritizing recent, high-impact publications.

Given the broad mechanistic scope of this structured narrative review, the literature synthesis also considered studies addressing immunometabolic mechanisms directly relevant to the predefined reproductive contexts, particularly chronic low-grade inflammation, insulin resistance, hyperandrogenemia, immune dysregulation, and oxidative stress. These mechanistic domains informed the thematic synthesis to ensure alignment between the evidence discussed and the objectives of the review.

**Figure 1:**
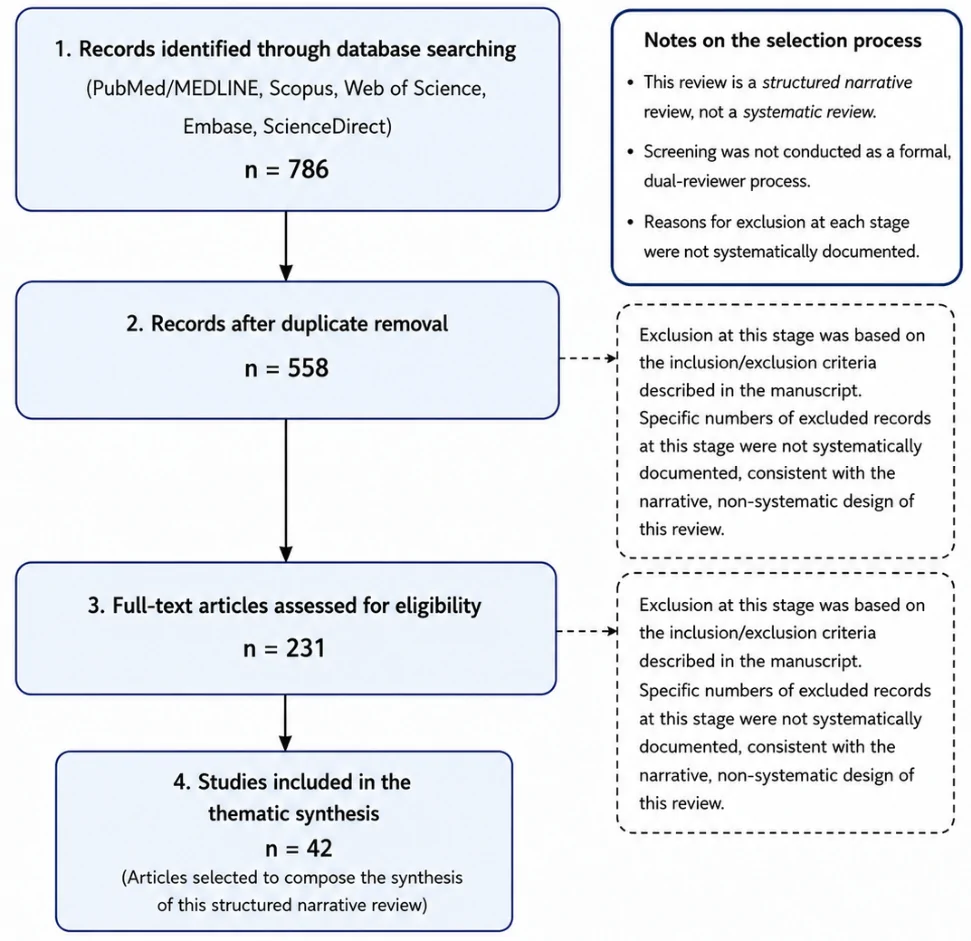
Literature selection process for the structured narrative review. The diagram summarizes the available screening records used to construct the review and should not be interpreted as PRISMA reporting or as representing a systematic-review methodology. Specific numbers of excluded records at each stage were not systematically documented, consistent with the narrative, non-systematic design of this review.

Inclusion criteria covered studies addressing obesity in women of reproductive age or presenting sex-stratified analyses, with emphasis on mechanisms affecting the ovary–endometrium–placenta axis. No formal language restriction was applied during the literature search. Studies focused exclusively on male populations, purely behavioral investigations lacking mechanistic analysis, and articles without full-text availability were excluded. Titles and abstracts identified through this search were screened for relevance, and the full texts of potentially eligible articles were then examined in greater detail to inform the thematic synthesis. Consistent with the narrative, non-systematic design of this review, this screening was not conducted as a formal, dual-reviewer process with a documented reason for exclusion at each stage. A descriptive flow diagram summarizing the study-selection process based on the available screening records is provided for transparency and visualization; it should not be interpreted as PRISMA reporting or as evidence of a systematic-review methodology (Figure 1). The body of literature ultimately selected for qualitative synthesis is discussed throughout the manuscript, in accordance with reporting considerations recommended for narrative reviews [8].

Data were analyzed descriptively and interpretively, using a thematic structure that moved from the general immunometabolic mechanisms of obesity toward its reported associations with ovarian, endometrial, and placental function. This approach allowed findings from heterogeneous study types, populations, and experimental models to be integrated into a conceptual synthesis, without implying a single, empirically confirmed causal pathway linking these outcomes within the same individual. Supplementary Table S1 summarizes, for each key reference, the study design or evidence type, population or experimental model, exposure or intervention where applicable, key findings relevant to this review, principal limitations, and relevance to the present synthesis. This classification allows the reader to distinguish, where applicable, among human, animal, in vitro, observational, experimental, and review-level evidence.

## 3. Pathophysiological Mechanisms

### 3.1. Visceral Adiposity and Chronic Low-Grade Inflammation

Female obesity is predominantly characterized by the accumulation of visceral adipose tissue, which shows high metabolic and secretory activity and acts as an active endocrine organ, releasing adipokines, chemokines, and pro-inflammatory cytokines, including tumor necrosis factor-alpha (TNF-α), interleukin-6 (IL-6), and C-reactive protein (CRP) [1,9]. This systemic inflammatory state extends beyond metabolism to reproductive tissues that are highly sensitive to inflammatory and hormonal signaling, namely the ovary, the endometrium, and the maternal–placental interface, providing the common substrate for the mechanisms discussed in the following sections [10].

### 3.2. Insulin Resistance and Hormonal Dysregulation

Compensatory hyperinsulinemia, arising from obesity-related insulin resistance (IR), acts directly on ovarian steroidogenesis, stimulating androgen production by theca cells and amplifying functional hyperandrogenemia. This mechanism, mediated by activation of intracellular pathways such as nuclear factor kappa B (NF-κB) and c-Jun N-terminal kinase (JNK), impairs insulin receptor substrate phosphorylation across multiple target tissues, including the ovary itself, a pattern of insulin-signaling impairment also documented in the broader context of diabetes-related reproductive dysfunction in women [11,12].

Functional hyperandrogenemia, in turn, disrupts the pulsatility of the hypothalamic–pituitary–ovarian axis, altering luteinizing hormone (LH) and follicle-stimulating hormone (FSH) secretion and favoring chronic anovulation. This hormonal imbalance is compounded by increased aromatase activity in expanded adipose tissue, which promotes peripheral conversion of androgens to estrogens and further destabilizes the reproductive axis [3,13].

Adipokine imbalance, marked by reduced adiponectin and elevated leptin, adds to this dysfunction of the hypothalamic–pituitary–ovarian axis. Chronically elevated leptin levels are associated with hypothalamic leptin resistance, disrupting pulsatile gonadotropin secretion and reinforcing the ovulatory dysfunction characteristic of female obesity [14].

This pathophysiological triad, compensatory hyperinsulinemia, functional hyperandrogenemia, and hypothalamic-pituitary-ovarian dysfunction, is most extensively documented in polycystic ovary syndrome (PCOS), and much of the mechanistic evidence discussed above derives specifically from PCOS populations or PCOS-model studies rather than from women with obesity without PCOS. Obesity should not be regarded as the primary cause of PCOS, but rather as a factor that may amplify its core mechanisms, intensifying IR, hyperandrogenemia, and anovulation in already predisposed women, with a more severe metabolic phenotype and greater resistance to ovulation induction protocols reported in some studies [9,15]. Because most available data do not consistently separate obesity-with-PCOS from obesity-without-PCOS, and because age, degree of obesity, and assisted reproductive technology (ART) status are additional sources of heterogeneity across the reviewed studies, these findings should not be generalized to all women with obesity; where the underlying literature does not allow this distinction, that limitation is acknowledged rather than inferred. Obesity and PCOS appear to share an overlapping immunometabolic substrate, and weight loss and reduced systemic inflammation are commonly proposed as first-line interventions for partially restoring ovulatory function, particularly in women with PCOS [16].

### 3.3. Ovarian Immunometabolic Dysfunction

The ovary is one of the sites most consistently implicated in obesity-related immunometabolic dysregulation, although a substantial portion of the underlying mechanistic evidence originates from studies in PCOS or aging populations rather than from obesity without PCOS specifically. Within the follicular microenvironment, the accumulation of inflammatory mediators, together with M1 macrophage infiltration and NLRP3 inflammasome activation, has been associated with follicular inflammation capable of disrupting multiple stages of folliculogenesis in these populations [17].

Granulosa cells, which are particularly sensitive to changes in the metabolic microenvironment, show mitochondrial dysfunction, reduced steroidogenic efficiency, and greater susceptibility to apoptosis in the context of obesity, undermining the metabolic and paracrine support required for proper oocyte maturation [18]. In this setting, oocyte quality is affected through converging pathways, including intraoocyte lipid accumulation, reduced adenosine triphosphate (ATP) production, increased reactive oxygen species (ROS), and redox imbalance, which together compromise meiotic competence, spindle organization, and genetic integrity, with downstream effects on fertilization rates and early embryonic competence [19]. Experimental studies further suggest changes in oocyte mitochondrial dynamics under conditions of obesity, although these specific molecular mechanisms still require broader confirmation in humans [18].

Taken together, these changes can be understood as a manifestation of broader metabolic overload and lipotoxicity within the ovarian microenvironment: excess lipid availability and impaired mitochondrial oxidative capacity in granulosa and cumulus cells disrupt redox homeostasis, and the resulting reactive oxygen species (ROS) accumulation has been implicated in impaired oocyte cellular integrity, mitochondrial function, and developmental competence [18,19]. This pattern is conceptually consistent with the oxidative and endothelial stress described at the maternal–placental interface (Section 3.5), although the two have not been shown to reflect a single, continuous mechanism within the same individual; each should be interpreted as a context-dependent manifestation of obesity-related redox dysregulation rather than as steps in a proven linear cascade.

Ovarian reserve, assessed through markers such as anti-Müllerian hormone (AMH) and antral follicle count, has been examined in relation to central adiposity, although the largest body of meta-analytic evidence to date describes this relationship as inconsistent and, in part, controversial across studies; where an inverse association is observed, it may still be of relevance for women pursuing fertility preservation or assisted reproduction [20]. Taken together, follicular inflammation, granulosa cell dysfunction, impaired oocyte quality, and diminished ovarian reserve converge into a picture of ovarian origin infertility whose mechanistic understanding is essential for more precise diagnostic and therapeutic strategies [17,18,20].

### 3.4. Endometrial Receptivity and Implantation Failure

Beyond its reported associations with ovarian function, obesity has also been associated with impaired endometrial receptivity, through disrupted decidualization of the endometrial stroma, a process that depends on coordinated hormonal signaling and a regulated immune microenvironment and is compromised by the chronic low-grade inflammation characteristic of obesity [10,21].

Implantation occurs within a tightly regulated window that depends on coordinated expression of adhesion molecules and transcription factors, most notably Homeobox A10 (HOXA10), essential for endometrial differentiation and uterine receptivity. In women with obesity and IR, HOXA10 and other adhesion-related genes are frequently downregulated, impairing early implantation and contributing to unexplained infertility and recurrent implantation failure, an effect that is more pronounced when obesity coexists with PCOS [22,23]. HOXA10 is itself a well-established regulator of endometrial receptivity, and post-translational mechanisms controlling its stability, such as SUMOylation, have been directly linked to recurrent implantation failure independently of body weight, underscoring its broader mechanistic role [24].

Leukemia inhibitory factor (LIF), a cytokine essential for blastocyst-endometrium signaling, is similarly altered in models of obesity, impairing embryo–endometrial communication and favoring pro-inflammatory cytokines (TNF-α, IL-6) over a tolerogenic microenvironment. This persistent inflammation has been linked to higher rates of implantation failure and early pregnancy loss, extending the reproductive impact of obesity beyond the ovarian axis [10,21,22].

Emerging transcriptomic evidence further implicates the endometrium itself as a site of obesity-related dysfunction. In infertile women with obesity and PCOS, endometrial RNA sequencing during the window of implantation has revealed dysregulated metabolic and inflammatory pathway genes compared with normal-weight controls, although this evidence derives specifically from a PCOS population and should not be generalized to obesity without PCOS [25]. Complementary in vitro data show that insulin exposure alone alters the endometrial epithelial transcriptome, affecting pathways involved in protein export, glutathione metabolism, and ribosomal function, suggesting a plausible mechanistic link between obesity-related hyperinsulinemia and endometrial gene expression independently of PCOS status [26].

Endometrial macrophages and uterine natural killer (uNK) cells further shape the local immune microenvironment relevant to implantation. Macrophages are abundant in the implantation-window endometrium and early decidua, and disordered polarization toward a pro-inflammatory phenotype has been associated with recurrent implantation failure; however, evidence linking obesity specifically to endometrial macrophage polarization in women remains indirect, derived largely from other patient populations or from adipose rather than endometrial tissue [27]. uNK cells similarly regulate decidualization, angiogenesis, and early trophoblast invasion [28] and show functional alterations in maternal obesity, as previously described in the placental context (Section 3.5) [29]; whether comparable changes occur earlier, at the level of the pre-conception endometrium, remains insufficiently studied in humans.

### 3.5. Placental Dysfunction and Preeclampsia

Once pregnancy is established, the same immunometabolic substrate that impairs the ovary and endometrium begins to act at the maternal–placental interface. Adequate placentation depends on extravillous trophoblast invasion and remodeling of the uterine spiral arteries into low resistance, high flow vessels [30]. In women with obesity, chronic systemic inflammation and IR have been associated with impaired trophoblast invasion and incomplete spiral artery remodeling, resulting in a persistently high resistance vascular bed, an early pathophysiological event repeatedly linked to preeclampsia [5,31]. Uterine natural killer (uNK) cells, whose function is essential for decidualization and vascular remodeling, also show functional alterations in maternal obesity, further contributing to this deficient trophoblast invasion [29].

An imbalance between pro- and anti-angiogenic factors represents another key element of this process. Reduced availability of vascular endothelial growth factor (VEGF) and placental growth factor (PlGF), together with elevated soluble fms-like tyrosine kinase-1 (sFlt-1), impairs placental angiogenesis and contributes to maternal systemic endothelial dysfunction, a mechanism widely recognized as central to the pathophysiology of preeclampsia [5].

The placentas of women with obesity also show greater macrophage infiltration and higher TNF-α and IL-6 expression, reflecting placental inflammation that adds to maternal systemic inflammation and amplifies oxidative stress at the maternal–fetal interface [4,32]. The resulting endothelial dysfunction is not confined to the uteroplacental bed, but extends to the maternal systemic circulation, contributing to the clinical features of preeclampsia, including hypertension, proteinuria, and, in severe cases, multiorgan dysfunction, such that maternal obesity not only raises the baseline risk of preeclampsia, but also appears to modulate its severity and time of onset [5,33].

Based on the evidence synthesized in this review, we propose a conceptual, hypothesis-generating model in which obesity-related preeclampsia may represent one possible downstream manifestation of immunometabolic disturbances that can also affect ovarian and endometrial function earlier in the reproductive process. This model does not imply that ovarian dysfunction and impaired endometrial receptivity necessarily precede or causally determine placental dysfunction within the same woman; rather, it reflects a set of mechanistically related, context-dependent associations drawn from heterogeneous populations and study designs, which future longitudinal research will need to test directly.

### 3.6. Integrated Immunometabolic Model

**Figure 2:**
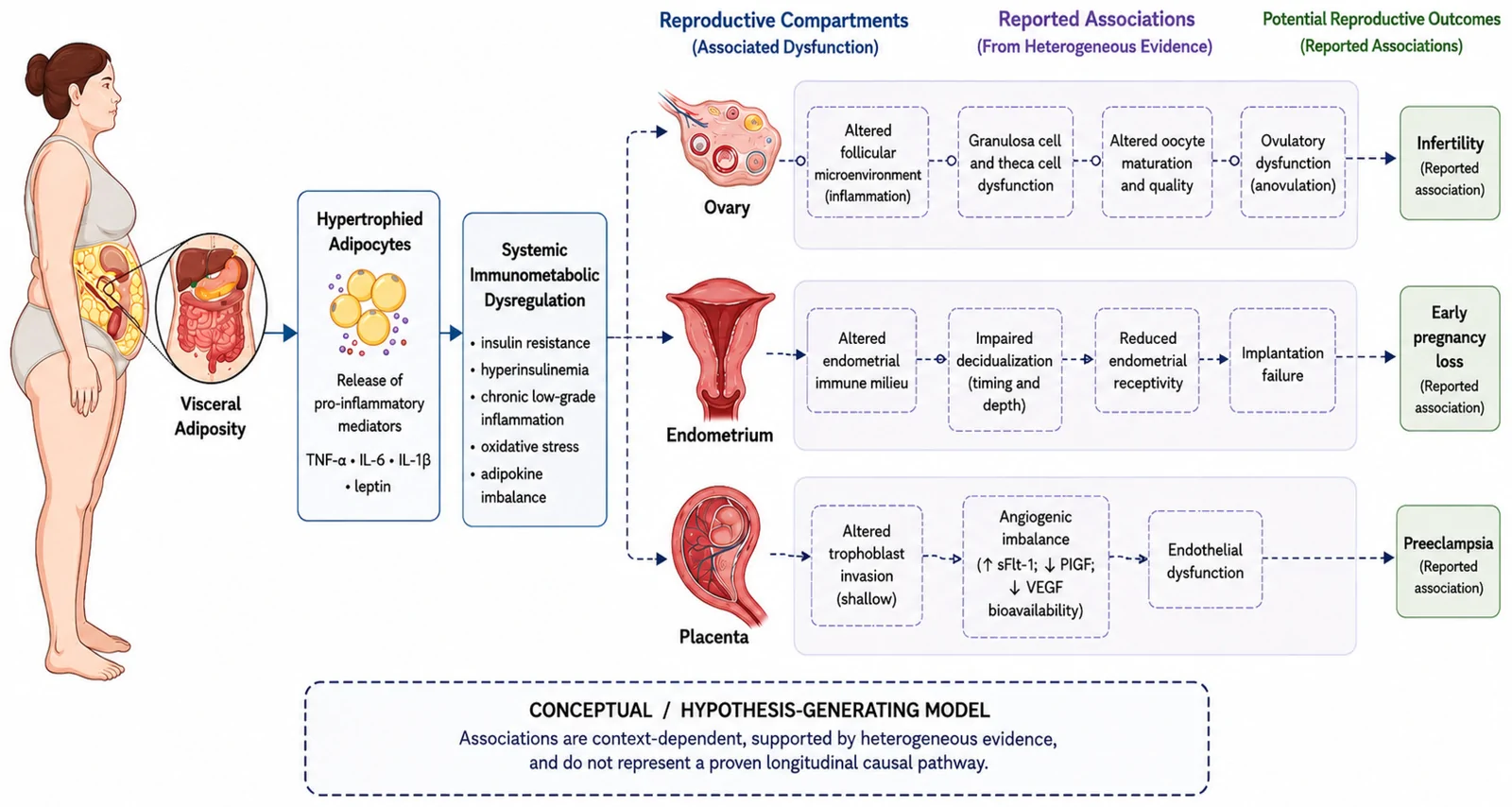
Conceptual, hypothesis-generating model of the immunometabolic mechanisms potentially linking maternal obesity to infertility and preeclampsia. Chronic low-grade systemic inflammation, insulin resistance, and functional hyperandrogenemia have been associated, in a context-dependent manner and with varying strength of evidence, with dysfunction in the ovary, endometrium, and placenta, which may contribute to infertility and preeclampsia. Dashed connectors indicate non-linear and non-sequential relationships. Figure generated with the assistance of Google Gemini (generative artificial intelligence) based on the mechanisms synthesized in this review and subsequently reviewed and edited by the authors for scientific accuracy. Note: This model synthesizes evidence from heterogeneous human studies and experimental models. Associations are context-dependent and not necessarily present in all individuals with obesity.

Taken together, these mechanisms support an integrated, conceptual immunometabolic model in which maternal obesity, systemic inflammation, insulin resistance, and functional hyperandrogenemia are proposed as interrelated contributors to ovarian dysfunction, impaired endometrial receptivity, and altered placentation, which may in turn be associated with infertility and preeclampsia, as illustrated in the hypothesis-generating framework in Figure 2.

Understanding these mechanisms as interrelated, rather than strictly isolated, supports the hypothesis that interventions targeting inflammation and maternal insulin resistance could have effects across more than one reproductive stage, although this remains to be confirmed prospectively. The main mechanisms and their reported associations with ovarian, endometrial, and placental function are summarized in Table 1, together with the corresponding level of evidence and key limitations.

## 4. Clinical Implications

The clinical implications of female obesity span the entire reproductive continuum, supporting an approach that moves beyond isolated anthropometric indices such as body mass index (BMI) to incorporate specific reproductive biomarkers. Ovarian reserve markers such as AMH, an established tool in assisted reproduction planning, combined with systemic inflammatory biomarkers such as CRP and IL-6, which remain largely investigational for this purpose, may eventually help identify women at greater risk of ovulatory dysfunction and reduced response to assisted reproduction protocols, pending further validation [20].

Obesity is associated with lower success rates across multiple stages of in vitro fertilization (IVF): reduced ovarian response requiring higher gonadotropin doses during stimulation [34]; the oocyte quality impairment discussed in Section 3.3, with lower clinical pregnancy and live birth rates as body mass index increases, at the laboratory stage [35]; and, at the transfer stage, the impaired endometrial receptivity described in Section 3.4, associated with lower implantation rates even with morphologically normal embryos [36]. The proportion of euploid embryos, however, does not appear to differ consistently across body mass index categories, so current evidence does not support higher embryonic aneuploidy in obesity. Together, these factors are associated with lower cumulative live birth rates per cycle in women with obesity, which supports further investigation of preconception metabolic optimization as a plausible, though not yet proven, component of assisted reproduction counseling [34,36].

Endometrial receptivity testing has been studied in selected cases of recurrent implantation failure using molecular assays, but its routine clinical value remains under evaluation, with no consistent benefit demonstrated in good-prognosis populations [37]. The potential role of angiogenic markers such as the sFlt-1/PlGF ratio for early risk stratification in pregnant women with obesity remains under investigation and should be distinguished from their more established diagnostic-support role when preeclampsia is clinically suspected [5,38].

Preconception interventions aimed at reducing inflammation and excess adiposity, including lifestyle changes, nutritional strategies, and physical activity, remain central to management, with potential benefits for ovulatory function, endometrial receptivity, and subsequent pregnancy outcomes [3,16].

Taken together, this evidence supports a more integrative clinical model that recognizes obesity as a systemic inflammatory condition potentially affecting the ovary–endometrium–placenta axis, incorporating metabolic, hormonal, reproductive, and obstetric dimensions into the care of women of reproductive age. The main biomarkers relevant to female reproduction, with potential application in screening and clinical monitoring, are summarized in Table 2.

## 5. Emerging Therapies and Future Directions

Recent advances in understanding the immunometabolic mechanisms of obesity have driven new therapeutic approaches aimed at improving female reproductive health. Glucagon-like peptide-1 (GLP-1) receptor agonists stand out as a promising preconception strategy, with consistent efficacy for weight loss and metabolic improvement and exploratory, unconfirmed signals of benefit for ovulatory function in women with obesity and PCOS. Their use should be confined to the preconception period, with a planned washout before attempting conception, since gestational safety has not been established [39,40,41].

Insulin sensitizers such as metformin remain a widely used option in women with obesity-related insulin resistance and ovulatory dysfunction, contributing to metabolic improvement and partial restoration of ovarian function, with potential benefits for assisted reproduction outcomes [41,42].

Modulating inflammation in adipose tissue and the reproductive microenvironment, including strategies targeting macrophage polarization (M1/M2) within the ovarian microenvironment, has shown promise in preclinical studies aimed at restoring reproductive homeostasis across the ovary–endometrium–placenta axis, although evidence to date derives from PCOS and preclinical models rather than obesity without PCOS [43].

Precision medicine applied to reproduction is also emerging, allowing identification of specific metabolic, ovarian, and endometrial profiles that could guide individualized protocols for ovarian stimulation, endometrial preparation, and obstetric surveillance [44]. Meaningful translational challenges remain, however, since identifying inflammatory and metabolic signatures with predictive value still depends on complex molecular analyses and technologies with limited accessibility, calling for further longitudinal studies to validate these strategies.

Interest in the gut microbiota, inflammation, and reproductive health is also growing, with evidence linking intestinal dysbiosis to systemic inflammation and ovulatory dysfunction and opening the door to microbiota-targeted interventions as a complementary preconception strategy [45,46]. Taken as a whole, these emerging approaches reinforce the idea that the future of managing female obesity in the reproductive context lies in integrated, multi-stage strategies, spanning preconception metabolic optimization through targeted obstetric monitoring.

The main emerging therapeutic strategies and their immunometabolic effects on reproductive health are summarized in Table 3.

## 6. Conclusions

Female obesity represents a complex immunometabolic condition associated with dysfunction across the ovary, endometrium, and placenta, potentially contributing to an increased risk of infertility and preeclampsia. A comprehensive understanding of these interrelated mechanisms supports a more integrated conceptual approach centered on the ovary–endometrium–placenta axis, one that moves beyond interpretations limited to excess body weight; incorporating and further validating specific reproductive biomarkers and early screening strategies may represent an important step forward in managing these patients.

Despite its broad scope, this review has limitations that should be acknowledged. Being a narrative review, it does not follow the formal methodological procedures of a systematic review or meta-analysis, such as dual independent screening, systematic documentation of reasons for exclusion at each stage, or a structured risk-of-bias assessment; this increases the potential for selection bias, and readers should interpret the synthesis accordingly. In addition, the included studies varied considerably in design, population characteristics, including inconsistent distinction between obesity with and without PCOS, and outcomes considered, which limits direct comparisons across findings and constrains the strength of the conceptual model proposed.

Even so, recognizing female obesity as an immunometabolic condition potentially associated with dysfunction across the ovary–endometrium–placenta axis supports continued research and reflection in reproductive clinical practice. Future work should prioritize longitudinal and translational studies capable of directly testing the proposed conceptual pathways and identifying validated biomarkers to improve early diagnosis and therapeutic monitoring. Ultimately, the findings from this review suggest that understanding female obesity through an immunometabolic lens may help inform more effective preventive and therapeutic strategies for reproductive health, pending confirmation by future studies.

## Figures and Tables

**Table 1:** Immunometabolic mechanisms in female obesity: primary reproductive target, main biological effect, downstream consequence, and evidence type/main limitation.

Mechanism	Primary Reproductive Target	Main Biological Effect	Downstream Consequence	Evidence Type/Main Limitation
Visceral adiposity and chronic low-grade inflammation	Multiple reproductive compartments (ovary, endometrium, placenta)	Systemic pro-inflammatory milieu (TNF-α, IL-6, CRP)	Associated with increased risk of infertility and preeclampsia [1,9]	Human observational and preclinical data; consistent direction; mostly cross-sectional, causality not established.
Insulin resistance and functional hyperandrogenemia	Ovary	Altered insulin signaling and steroidogenesis (anovulation, hyperandrogenemia)	Associated with ovulatory dysfunction and infertility [11,12]	Human and experimental data, largely from PCOS-enriched samples; consistent direction; limited direct data in obesity without PCOS.
Exacerbation of polycystic ovary syndrome	Ovary	Amplification of the hyperandrogenic/metabolic PCOS phenotype	Associated with reduced response to fertility treatments [15]	Human observational data, mainly in PCOS populations; consistent direction; evidence specific to PCOS, not generalizable to obesity without PCOS.
Follicular inflammation and granulosa cell dysfunction	Ovary	Follicular inflammatory signaling; granulosa cell dysfunction	Associated with reduced oocyte quality [17,18]	Mainly preclinical and PCOS-model data; consistent direction; limited confirmation in obesity without PCOS.
Oocyte oxidative stress	Ovary (oocyte)	Mitochondrial/redox dysfunction (ROS accumulation)	Associated with reduced embryonic developmental competence [19]	Mainly experimental/animal data; consistent direction; human confirmation still limited.
Reduced ovarian reserve	Ovary	Decreased follicular reserve (reduced AMH)	Potentially linked to reduced response to assisted reproductive technologies [20]	Human observational data (meta-analysis level); heterogeneous/inconsistent association; causality not established.
Impaired decidualization and endometrial receptivity	Endometrium	Impaired decidualization and receptivity (inflammatory signaling, HOXA10 dysregulation)	Associated with implantation failure and early pregnancy loss [21,22,24,25,26]	Human transcriptomic and in vitro data; consistent direction; small samples, mechanistic causality unproven.
Impaired trophoblast invasion	Placenta	Superficial placentation; defective spiral artery remodeling	Consistent with an early pathogenic event in preeclampsia [30,31]	Human placental and preclinical data; consistent direction; associative, not longitudinal.
Angiogenic imbalance	Placenta	Angiogenic imbalance (VEGF/PlGF/sFlt-1 dysregulation)	Associated with hypertension and proteinuria [5]	Human clinical data, well validated for preeclampsia; consistent direction; validated mainly for risk assessment, not general obesity screening.
Placental inflammation	Placenta	Placental inflammatory/oxidative signaling	Associated with increased severity and earlier onset of preeclampsia [4,32,33]	Human placental observational data; consistent direction; associative, underlying mechanism not fully resolved.

Source: Developed by the authors based on the reviewed literature. Abbreviations: TNF-α, tumor necrosis factor-alpha; IL-6, interleukin-6; CRP, C-reactive protein; ROS, reactive oxygen species; AMH, anti-Müllerian hormone; HOXA10, Homeobox A10; PCOS, polycystic ovary syndrome; VEGF, vascular endothelial growth factor; PlGF, placental growth factor; sFlt-1, soluble fms-like tyrosine kinase-1.

**Table 2:** Clinical biomarkers and their application in the reproductive assessment of women with obesity, with an indication of their current level of clinical validation.

Biomarker/Clinical Parameter	Pathophysiological Association	Main Clinical Implications	Current Level of Clinical Validation
Body mass index (BMI) and waist circumference	Visceral adiposity	Increased risk of infertility and obstetric complications	Established initial reproductive risk assessment; low specificity for individual mechanisms [1]
C-reactive protein (CRP)	Chronic systemic inflammation	Increased risk of ovulatory dysfunction and preeclampsia	Investigational for reproductive risk stratification; not established as a routine screening biomarker in women with obesity [20]
IL-6	Systemic and placental inflammation	Ovarian, endometrial, and endothelial dysfunction	Investigational; used mainly in research settings, not established for routine clinical monitoring [4]
Anti-Müllerian hormone (AMH) and antral follicle count	Reduced ovarian reserve	Reduced response to ovarian stimulation	Established for planning of assisted reproductive technology protocols [20]
Androgens (total/free testosterone)	Functional hyperandrogenemia/PCOS	Anovulation and infertility	Established complementary hormonal assessment, mainly in suspected PCOS [15]
HOMA-IR	Insulin resistance	Ovulatory dysfunction and worsening of PCOS	Commonly used research and clinical tool; supportive rather than diagnostic role in candidate selection for insulin-sensitizing therapy [11]
Endometrial receptivity markers (HOXA10, LIF)	Impaired decidualization	Recurrent implantation failure	Investigational; not recommended for routine clinical use outside research protocols [22,37]
sFlt-1/PlGF ratio	Placental angiogenic imbalance	Early prediction and diagnostic support for preeclampsia	Validated for diagnostic support once preeclampsia is clinically suspected; use for general screening of women with obesity remains investigational [38]
Reduced adiponectin/elevated leptin	Adipokine dysregulation	Dysfunction of the hypothalamic–pituitary–ovarian axis	Investigational; not yet part of standardized clinical assessment [14]

Source: Developed by the authors based on the reviewed literature. Abbreviations: BMI, body mass index; CRP, C-reactive protein; IL-6, interleukin-6; AMH, anti-Müllerian hormone; PCOS, polycystic ovary syndrome; HOMA-IR, Homeostatic Model Assessment for Insulin Resistance; HOXA10, Homeobox A10; LIF, leukemia inhibitory factor; sFlt-1, soluble fms-like tyrosine kinase-1; PlGF, placental growth factor.

**Table 3:** Therapeutic and management strategies for obesity-related reproductive dysfunction, organized by evidence level and clinical status.

Therapeutic Strategy	Mechanism of Action	Immunometabolic/Reproductive Effects	Potential Clinical Benefits	Evidence Level/Clinical Status
Preconception lifestyle interventions	Reduction of adiposity and systemic inflammation	Improved ovulatory function and endometrial receptivity	Metabolic optimization before assisted reproductive treatment [3,16]	Established/recommended for obesity and metabolic health; supports ovulatory and endometrial benefit, but not proof of causally improved live birth
Metabolic optimization before assisted reproduction	Structured weight reduction before IVF	Association with improved ovarian response and embryo quality suggested in observational data; effect on implantation and live birth not yet demonstrated in interventional studies	Currently supported mainly by evidence that obesity is associated with lower live birth rates, not by interventional proof that pre-IVF weight loss increases cumulative live birth [34,36]	Established for metabolic/weight optimization; reproductive-context benefit currently supported only by association data, not interventional proof
Insulin sensitizers (metformin)	Reduction of insulin resistance	Partial restoration of ovulatory function	Adjunctive therapy for ovulation induction and assisted reproductive technologies [41,42]	Clinically used in selected patients (insulin resistance/PCOS); not a universal treatment for obesity-related infertility
GLP-1 receptor agonists	Weight reduction and metabolic improvement	Improved metabolic profile and preliminary evidence of enhanced ovulatory function; restricted to the preconception period	Metabolic optimization in women with obesity and PCOS [39,40,41]	Emerging/insufficient evidence for reproductive outcomes; established metabolic benefit, periconception safety not established
Modulation of macrophage polarization (M1/M2)	Reduction of ovarian and endometrial inflammation	Restoration of immune homeostasis	Preclinical strategy with translational potential [43]	Emerging/preclinical; predominantly experimental, insufficient evidence for clinical reproductive use
Gut microbiota modulation	Reduction of dysbiosis and systemic inflammation	Potential improvement in ovulatory function	Emerging complementary therapeutic strategy [45,46]	Emerging/investigational; insufficient evidence for reproductive outcomes
Precision reproductive medicine	Identification of individualized metabolic and reproductive profiles	Personalized therapeutic protocols	Currently limited by cost and technological complexity [44]	Emerging/future perspective; not yet an established clinical intervention

Source: Developed by the authors based on the reviewed literature. Abbreviations: GLP-1, glucagon-like peptide-1; PCOS, polycystic ovary syndrome; IVF, in vitro fertilization.

## Data Availability

No new data were created or analyzed in this study. Data sharing is not applicable to this article.

## Supplementary Materials

The following supporting information can be downloaded at: https://www.mdpi.com/article/10.3390/biomedicines14092039/s1, Table S1: Characteristics, evidence type, and critical appraisal of the literature informing the narrative synthesis. References [1,2,3,4,5,6,7,8,9,10,11,12,13,14,15,16,17,18,19,20,21,22,23,24,25,26,27,28,29,30,31,32,33,34,35,36,37,38,39,40,41,42,43,44,45,46] are cited in Supplementary Materials.
