# Supplementary material for: Immunometabolic Dysregulation in Female Obesity: Mechanistic Links to Ovarian Dysfunction, Implantation Failure, and Preeclampsia
Source: Biomedicines. 2026 Sep 10;14(9):2039. doi: 10.3390/biomedicines14092039 (PMC13604285; doi:10.3390/biomedicines14092039)
Supplement: Supplementary file 1 [file biomedicines-14-02039-s001.zip › biomedicines-4511520-supplementary.pdf]

Supplementary Table S1. Characteristics, evidence type, and critical appraisal of the literature informing the narrative synthesis.

Note: The literature informing this narrative synthesis includes different levels and types of evidence (systematic reviews and meta-analyses, primary human observational and experimental studies, animal and in vitro studies, clinical guidelines, and narrative reviews). Studies conducted specifically in populations with polycystic ovary syndrome (PCOS) are identified explicitly and were not extrapolated as evidence for obesity independent of PCOS. Experimental/preclinical evidence (animal or in vitro) was not treated as equivalent to clinical evidence. The heterogeneity of study designs summarized below limits causal inference across the ovary-endometrium-placenta axis discussed in the main text.

Ref	Reference	Study design / evidence type	Population / experimental model	Exposure / intervention / topic	Key finding(s) relevant to this review	Main limitations	Relevance to the present review
1	Šišljagić et al., 2024, Biomedicines 12(12):2801	Narrative review	General literature on uterine physiology and obesity (no single study population)	Not applicable	Obesity-related insulin and leptin resistance alter uterine morphology, hormonal signaling, and inflammatory processes affecting reproductive outcomes	Narrative review; no systematic search methodology reported	General background reference for obesity's impact on uterine/reproductive function
2	Yong et al., 2023, Int J Med Sci 20(3):366-375	Narrative review	General literature on obesity and female reproduction	Not applicable	Obese women show lower gonadotropin levels, reduced fecundity, higher miscarriage rates, and poorer IVF outcomes; adipose tissue immune cells drive chronic low-grade inflammation	Narrative review; no systematic search/selection methodology reported	General background reference for obesity's impact on reproductive dysfunction
3	Malhotra et al., 2025, Cureus 17(7):e87283	Narrative review (explicit PubMed search strategy, 1995-2025, reported in methods)	Women of reproductive age with obesity	Weight-loss interventions (lifestyle, GLP-1RAs, bariatric surgery) vs no intervention, across included studies	Obesity disrupts the HPO axis, oocyte quality/reserve, and endometrial receptivity; weight-loss interventions show promise for anovulation but variable/inconsistent impact on live birth rates	Narrative (non-systematic) review; authors note reliance on animal/in vitro data for mechanistic sections	Supports obesity's impact on fertility and the first-line role of weight loss/anti-inflammatory strategies
4	Ramanlal et al., 2026, Mol Hum Reprod 32(1):gaag002	Narrative review	General literature on maternal obesity and placental vasculature (no single study population)	Not applicable	In maternal obesity, the placenta shows lipotoxicity, inflammation, and cellular stress that may impair angiogenic signalling, endothelial metabolism, and vascular development	Narrative review; mechanisms drawn largely from preclinical/experimental placental studies	Cited for obesity's impact on placental vascular development and placental inflammation
5	Lopez-Jaramillo et al., 2018, Front Physiol 9:1838	Narrative/mini review	General literature on obesity and preeclampsia across populations	Not applicable	Abnormal placentation/impaired trophoblast invasion and spiral artery remodeling precede clinical preeclampsia; obesity-related hyperinsulinism, IR, and systemic inflammation proposed as contributing mechanisms	Narrative review synthesizing heterogeneous studies; mechanisms not tested within a single cohort	Core reference for shared obesity-preeclampsia mechanisms
6	Sukhera, 2022, J Grad Med Educ 14:414-417	Methodological/guidance article — not part of the clinical/mechanistic evidence synthesis	Not applicable	Not applicable	Provides guidance on conducting and reporting narrative reviews	Not applicable	Cited to justify the structured narrative review design
7	Hosseini et al., 2024, J Med Surg Public Health 2:100046	Methodological/guidance article — not part of the clinical/mechanistic evidence synthesis	Not applicable	Not applicable	Guidance on formulating research questions for evidence-based studies (e.g., PICo framework)	Not applicable	Cited to justify the PICo-framed research question
8	Baethge et al., 2019, Res Integr Peer Rev 4:5	Methodological article — not part of the clinical/mechanistic evidence synthesis	Not applicable	Not applicable	Presents the SANRA scale for quality assessment of narrative review articles	Not applicable	Cited to justify the reporting approach for this narrative review
9	Deng et al., 2024, Front Immunol 15:1470283	Narrative review	Women with PCOS (obesity/IR discussed as contributing factors, not as the primary study population)	Not applicable	Immune cell imbalance and pro-inflammatory cytokines (IL-6, TNF-alpha) drive systemic low-grade chronic inflammation in PCOS, affecting reproductive and other	Narrative review; evidence specific to PCOS — should not be generalized to obesity without PCOS	Used to support the IR/hyperandrogenemia/PCOS inflammatory triad

Ref	Reference	Study design / evidence type	Population / experimental model	Exposure / intervention / topic	Key finding(s) relevant to this review	Main limitations	Relevance to the present review
					organ systems		
10	Gonnella et al., 2024, Int J Mol Sci 25:6855	Narrative review	General molecular/experimental literature on obesity and the endometrium (no single cohort)	Not applicable	Obesity-related adipose tissue expansion modulates endometrial gene-expression pathways, creating a hostile endometrial environment that impairs uterine receptivity and implantation	Narrative review of molecular/experimental literature; mechanisms not tested in a single controlled cohort	Cited on the molecular link between obesity and the endometrial environment
11	Ding et al., 2021, Front Endocrinol 12:741764	Review (journal-labelled)	Women with PCOS	Not applicable	Hyperandrogenemia and insulin resistance proposed as the two major underlying causes initiating PCOS and its severity/duration	Narrative review; PCOS-specific evidence, not obesity in general	Cited on insulin resistance and elevated androgens as PCOS mechanisms
12	Thong et al., 2020, Lancet Diabetes Endocrinol 8(2):134-149	Clinical/narrative review	Women with diabetes (type 1 and type 2) — NOT an obesity-specific population	Not applicable	Reproductive dysfunction is a common but understudied complication of diabetes; reviews effects across the HPO axis and insulin signaling	Narrative review; primary focus is diabetes — extrapolation to obesity-related IR is indirect	Cited on IR-related impairment of insulin receptor signaling across tissues (population mismatch: diabetes, not obesity, noted as inconsistency)
13	Kuryłowicz, 2023, Biomedicines 11(3):690	Narrative review	General literature on estrogen and adipose tissue physiology (evidence base described by the author as largely preclinical)	Not applicable	Estrogen has a bidirectional relationship with adipose tissue; deficiency promotes fat accumulation, and obesity alters estrogen receptor/enzyme expression in adipose tissue	Narrative review; author-acknowledged reliance on preclinical (non-human) studies for mechanistic claims	Cited on aromatase activity and peripheral androgen-to-estrogen conversion
14	Zheng et al., 2024, Front Endocrinol 14:1326546	Mini review (journal-labelled)	General literature on obesity and female reproductive health	Not applicable	Obesity affects female reproductive health broadly (menstrual cycles, fertility, pregnancy outcomes); adipokine dysregulation implicated	Mini review format; narrative synthesis, not systematic	Cited on adipokine imbalance and hypothalamic-pituitary-ovarian dysfunction
15	Barber & Franks, 2021, Clin Endocrinol 95(4):531-541	Narrative review	Women with obesity and PCOS	Not applicable	Rising global obesity prevalence has driven increased PCOS prevalence; discusses pathogenesis and clinical management implications	Narrative review; PCOS-specific population, not generalizable to obesity without PCOS	Cited on obesity as an amplifier of PCOS mechanisms and on androgen assessment
16	Teede et al., 2023, J Clin Endocrinol Metab 108(10):2447-2469	Clinical practice guideline (international, evidence-based; 254 recommendations)	Women with PCOS (international multidisciplinary panel plus consumer input)	Various PCOS management strategies (lifestyle, pharmacological, etc.) appraised across the evidence base	Provides 254 evidence-based recommendations/practice points for PCOS assessment and management, including weight/lifestyle strategies as first-line	Guideline synthesis (not primary data); PCOS-specific	Cited to support weight loss/anti-inflammatory strategies as first-line PCOS interventions
17	Orisaka et al., 2023, Front Endocrinol 14:1324429	Narrative review	Women with PCOS, endometriosis, and aging (three distinct conditions compared); the obesity+PCOS-specific mechanism is one subset discussed	Not applicable	Chronic low-grade inflammation disrupts the ovarian microenvironment via distinct mechanisms in PCOS, endometriosis, and aging; in obesity+PCOS specifically, hyperandrogenemia/IR induce ovarian inflammation	Narrative review; the follicular inflammation/M1 macrophage/NLRP3 mechanism is specific to PCOS/endometriosis/aging populations, not obesity alone (inconsistency noted)	Cited on follicular inflammation, M1 macrophage infiltration, and NLRP3 inflammasome activation
18	Gao et al., 2023, Front	Review (journal-	Women with PCOS	Not applicable	Oxidative stress and mitochondrial	Narrative review; PCOS-	Cited on granulosa

Ref	Reference	Study design / evidence type	Population / experimental model	Exposure / intervention / topic	Key finding(s) relevant to this review	Main limitations	Relevance to the present review
	Med 10:1193749	labelled)	(obesity listed only as a clinical manifestation of PCOS, not the primary exposure)		dysfunction in granulosa cells are significantly higher in PCOS vs healthy controls, contributing to impaired follicular development	specific population, not obesity-without-PCOS	cell mitochondrial dysfunction and oxidative stress
19	Heng et al., 2024, Anim Res One Health 2(4):377-399	Narrative review	General literature on obesity and female fertility (human-focused topic; authors affiliated with animal-science institutes, but the article itself is a review, not a primary animal experiment)	Not applicable	Obesity-induced oxidative stress affects follicular atresia, oocyte maturation, and embryo implantation; mitochondrial lipid metabolism/antioxidant pathways discussed as therapeutic targets	Narrative review; authors state mechanistic understanding of obesity-induced oxidative stress in female reproduction 'remains limited'	Cited on oocyte oxidative stress mechanisms (lipid accumulation, ROS, redox imbalance)
20	Moslehi et al., 2018, Menopause 25(9):1046-1055	Systematic review and meta-analysis	Reproductive-aged women; analyses stratified by PCOS and fertility status	Not applicable	IMPORTANT: the authors' own abstract describes the association between BMI/obesity and ovarian reserve as 'controversial' — the meta-analysis was conducted precisely because prior evidence was inconsistent	Heterogeneous original studies pooled; overall relationship described by the authors as controversial, not a settled inverse association	Supports a cautious interpretation of the association between BMI/obesity and ovarian reserve, given heterogeneous and inconsistent evidence.
21	Metwally et al., 2014, Reprod Biol Endocrinol 12:75	Primary human observational study (proteomic tissue analysis)	16 women with recurrent miscarriage (obese, n=12; lean, n=4) and 5 fertile volunteers (obese, n=2; lean, n=3); midluteal-phase endometrial samples	Obesity/overweight (obese vs lean comparison)	Obese/overweight women with recurrent miscarriage showed significantly increased endometrial expression of haptoglobin (an inflammatory marker), transthyretin, and beta-globulin compared with lean counterparts; principal component analysis showed endometrial protein profiles clustering by BMI	Small sample; preliminary/exploratory proteomic study; authors describe findings as preliminary evidence only	Supports altered endometrial inflammatory/immune protein expression associated with obesity, relevant to implantation failure and early pregnancy loss.
22	Schulte et al., 2015, Reprod Sci 22(1):6-14	Narrative review	Women with obesity AND PCOS (both conditions jointly discussed)	Not applicable	Obesity and PCOS both drive compensatory hyperinsulinemia affecting endometrial glucose metabolism/homeostasis required for implantation-window preparation	Narrative review; does not disentangle obesity-only vs PCOS-only effects	Cited on metabolic derangements affecting endometrial receptivity at implantation
23	Contessa et al., 2026, Obes Endocrinol 2(1):wjag003	Review article (journal-labelled)	General reproductive-age population (energy metabolism/reproduction linkage), not a single cohort	Not applicable	Reproductive function is intrinsically linked to body metabolism; both energy deficiency and excess affect pubertal onset and fertility	Narrative review	Cited alongside ref. 22 on obesity and infertility
24	Jiang et al., 2017, Cell Death Discov 3:17057	Translational/mechanistic study (in vitro Ishikawa/BeWo cells, mouse embryo attachment assay, human endometrial tissue comparison)	Women with recurrent implantation failure (RIF) vs fertile controls undergoing IVF-ET; obesity/BMI status NOT examined in this study	Not applicable (mechanistic, not an intervention study)	SUMOylation of HOXA10 at lysine-164 reduces its protein stability/transcriptional activity and is elevated in RIF endometria, impairing embryo attachment in vitro	In vitro/animal-model mechanistic study; no obesity/BMI data collected	Supports the mechanistic role of HOXA10 dysregulation in impaired endometrial receptivity and implantation; not obesity-specific.
25	Szukiewicz et al., 2025, Int J Mol Sci	Narrative review	General mechanistic literature on trophoblast	Not applicable	Reviews mechanosensing of shear stress by extravillous trophoblasts	Narrative review; not specific to maternal	General trophoblast/placenta

Ref	Reference	Study design / evidence type	Population / experimental model	Exposure / intervention / topic	Key finding(s) relevant to this review	Main limitations	Relevance to the present review
	26(19):9565		biology in early pregnancy; obesity not mentioned		during uterine spiral artery remodeling	obesity	tion mechanism reference, not obesity-specific
26	Hoch et al., 2024, Mol Hum Reprod 30(8):gaae027	Primary human observational/experimental study (placental tissue analysis plus in vitro TNF-alpha explant experiments)	Lean vs obese non-smoking pregnant women, first-trimester placental tissue (4-12 gestational weeks)	Obesity (lean vs obese comparison); TNF-alpha treatment in vitro	Obesity associated with higher DNA damage (COMET assay) in first-trimester placenta, reduced DNA repair gene expression, increased villous cytotrophoblast apoptosis, and reduced proliferation	Human observational comparison plus in vitro explant experiments; causal link to clinical outcomes not established; first-trimester tissue only	Cited on obesity-associated genotoxic stress and altered trophoblast turnover
27	Perdu et al., 2016, JCI Insight 1(11):e85560	Primary human observational study (cohort comparison) with mechanistic gene-expression analysis	Cohort of obese or lean pregnant women (human uterine NK cell samples)	Obesity (obese vs lean comparison)	Obesity associated with significantly reduced uNK cell numbers and impaired uterine artery remodeling; obese-derived uNK cells hyper-responsive to PDGF, overexpressing decorin, inhibiting trophoblast survival in vitro	Observational cohort comparison; sample size not specified in the available abstract; mechanistic follow-up in vitro	Cited on functional alterations of uterine NK cells in maternal obesity
28	St-Germain et al., 2020, Int J Mol Sci 21(11):3776	Narrative review	General literature on maternal obesity and uterine immune cells, not a single cohort	Not applicable	Reviews evidence that maternal obesity and obesity-associated chronic low-grade inflammation shape uterine immune cell function, potentially underlying obesity-related pregnancy disorders	Narrative review	Cited on placental macrophage infiltration and inflammation
29	Chen et al., 2026, J Clin Hypertens 28(1):e70205	Prospective birth cohort study	21,893 singleton pregnant women, China	Pre-pregnancy BMI-based obesity phenotype combined with metabolic health status	Interaction between obesity phenotype and metabolic health significantly affects risk of gestational hypertension and preeclampsia (metabolically unhealthy overweight/obese = highest risk)	Observational cohort (China-specific population); generalizability to other populations not established; residual confounding possible	Cited on the interaction between maternal obesity and metabolic dysfunction in gestational hypertension/preeclampsia
30	Sermondade et al., 2019, Hum Reprod Update 25(4):439-451	Systematic review and meta-analysis	Women with obesity (BMI >30) undergoing IVF	Not applicable	Female obesity negatively and significantly impacts live birth rates following IVF; whether weight loss reverses this effect requires further study	Heterogeneity of included studies in population, group definitions, and outcomes; authors state this 'prevents drawing firm conclusions'	Supports the association between female obesity and reduced live birth rates following IVF.
31	Provost et al., 2016, Fertil Steril 105(3):663-669	Retrospective registry cohort study (239,127 autologous IVF cycles, SART registry 2008-2010)	Women undergoing autologous IVF in the US (SART registry)	BMI category (continuous/categorical)	Pregnancy/live birth outcomes decline as BMI increases	Registry/observational data; residual confounding; no data on weight-loss intervention effects	Supports the decline in pregnancy and live birth outcomes with increasing BMI in a large autologous IVF registry cohort.
32	García-Ferreyra et al., 2021, JBRA Assist Reprod 25(3):394-402	Retrospective, nonrandomized cohort study	191 IVF/ICSI cycles (non-donor oocytes) grouped by BMI: normal (n=67), overweight (n=86), obesity (n=38); Guayaquil, Ecuador	Overweight/obesity (BMI category) vs normal BMI	Overweight/obesity groups had lower blastocyst development rate and significantly lower pregnancy, implantation, and live birth rates vs normal-BMI group; fertilization rate and D3 embryo quality were similar across groups	Retrospective, nonrandomized; modest sample especially in the obesity subgroup (n=38); single-center	Supports an association between overweight/obesity and lower IVF outcomes, without establishing that pre-IVF weight loss improves these outcomes.
33	Liu et al., 2022, Front	Systematic review and	Infertile women	Endometrial	WOI displacement estimated in ~34-	Meta-analysis of	Consistent with

Ref	Reference	Study design / evidence type	Population / experimental model	Exposure / intervention / topic	Key finding(s) relevant to this review	Main limitations	Relevance to the present review
	Physiol 13:841437	meta-analysis (PROSPERO-registered)	undergoing IVF/ICSI (multiple pooled studies)	receptivity array (ERA)-guided personalized embryo transfer vs routine embryo transfer	38% of infertile women (RIF or good-prognosis groups); no significant difference in ongoing pregnancy/live birth rate between routine and ERA-guided personalized embryo transfer overall	heterogeneous studies	manuscript's statement of 'no consistent benefit... in good prognosis populations'
34	Stepan et al., 2023, Ultrasound Obstet Gynecol 61(2):168-180	Review article (journal-labelled)	Pregnant women (singleton and twin) screened/monitored for preeclampsia and fetal growth restriction; synthesizes multiple validation studies	sFlt-1/PlGF ratio testing	PlGF, sFlt-1, and the sFlt-1/PlGF ratio are useful for screening, diagnosing, predicting, and monitoring placenta-related disorders; authors propose integrating these tests into routine management of suspected preeclampsia/FGR	Narrative review; validated primarily for suspected/established preeclampsia, not for general screening of obese women without other risk indicators	Supports the manuscript's distinction between early predictive use and validated diagnostic use
35	Cena et al., 2020, J Clin Endocrinol Metab 105(8):e2695-2709	Mini-review (journal-labelled)	Women with obesity/overweight and PCOS	GLP-1 receptor agonists (as an emerging therapeutic option)	Reviews mechanisms of obesity/PCOS-related infertility and the emerging role of GLP-1RAs; states weight reduction is the most significant factor affecting fertility outcomes	Narrative review; editorial assistance for this article was funded by Novo Nordisk (a GLP-1RA manufacturer) — a potential conflict-of-interest consideration	Cited on GLP-1RAs as an emerging preconception strategy
36	Song et al., 2026, Front Nutr 13:1809416	Narrative review with a proposed decision-framework (evidence synthesis, not primary data)	Women with PCOS (metabolic and reproductive)	GLP-1 receptor agonists	GLP-1RAs address metabolic drivers of PCOS but fertility-focused evidence remains inconsistent due to limited reproductive endpoint data; authors propose a Bayesian-informed framework integrating RCT metabolic-outcome evidence with exploratory reproductive-outcome findings	Authors state 'limited reproductive endpoint data' and periconception safety concerns; framework is interpretive, not new trial data	Cited on balancing metabolic optimization and reproductive safety with GLP-1RAs
37	Hoteit et al., 2025, Ther Adv Endocrinol Metab 16:20420188251383064	Narrative review (explicitly reviews both human and animal trial data)	Women with PCOS (human clinical + animal model data reviewed)	GLP-1 receptor agonists	Reviews the dual metabolic and reproductive impact of GLP-1 receptor agonists in PCOS	Narrative review combining human and animal evidence; animal findings should not be treated as directly generalizable to humans	Cited on the dual metabolic/reproductive impact of GLP-1RAs in PCOS
38	Han et al., 2019, Reprod Biomed Online 39(2):332-342	Systematic review and meta-analysis (8 RCTs)	Women with PCOS (obese subgroup highlighted)	GLP-1 receptor agonists vs metformin	GLP-1RAs more effective than metformin at reducing BMI/abdominal girth and improving insulin sensitivity in PCOS; evidence graded moderate-to-low quality; authors state findings are 'inconclusive'	Only 8 RCTs pooled; authors explicitly note evidence quality is moderate-to-low	Cited on comparison of GLP-1RAs versus metformin in PCOS
39	Salehi et al., 2023, Front Immunol 14:1104550	Primary experimental/translational study (human PCOS follicular-fluid comparison, rat ovarian model, in vitro macrophage/granulosa-cell experiments)	Human PCOS vs non-PCOS subjects (follicular M1/M2 ratio), plus rat model and in vitro experiments; obesity not specifically addressed	Androgen exposure (endogenous, PCOS context); miR-379-5p overexpression/inhibition (experimental)	PCOS subjects had higher M1/M2 macrophage ratio than non-PCOS; granulosa-cell-derived exosomal miR-379-5p (androgen-induced) drives M1 macrophage polarization in preantral follicles	Mixed human/animal/in vitro design; mechanism demonstrated in PCOS, not obesity specifically	Cited on macrophage polarization (M1/M2) modulation via granulosa cell-derived miR-379-5p — should not be generalized to obesity without

Ref	Reference	Study design / evidence type	Population / experimental model	Exposure / intervention / topic	Key finding(s) relevant to this review	Main limitations	Relevance to the present review
							PCOS
40	Zhang & Yu, 2020, Front Cell Dev Biol 7:382	Review (journal-labelled)	Not a clinical population — reviews precision medicine applications in reproductive medicine and gynecologic cancer broadly; obesity not mentioned	Not applicable	Precision medicine (large-scale genetic data, knowledge discovery) is being applied to diagnosis, treatment planning, and embryo identification in reproductive medicine and gynecologic cancer	Narrative review; authors themselves note the field's limitations and challenges 'remain to be discussed'; not specific to obesity	Cited on precision medicine approaches applicable to reproductive care
41	Balagoni et al., 2026, Health Sci Rep 9:e72399	Narrative review (explicit in title)	Women with infertility (general); gut microbiota focus, linked to PCOS, endometriosis, ovulatory dysfunction, pregnancy complications	Probiotics, dietary modification, weight management, lifestyle interventions (across included studies)	Dysbiosis is linked to multiple reproductive conditions; probiotics/diet/weight management/lifestyle show promise but clinical role remains uncertain due to study heterogeneity	Narrative review; authors state clinical role 'remains uncertain' due to heterogeneous study designs	Cited on the bidirectional relationship between gut microbiota and female infertility
42	Zhang et al., 2025, Front Nutr 12:1583805	Cross-sectional study (NHANES 2013-2018)	3,053 U.S. women aged 18-45 (NHANES database)	Dietary index for gut microbiota (DI-GM), a dietary-pattern-derived score	Association between DI-GM and female infertility examined in a nationally representative U.S. sample	Cross-sectional design (no causal inference); self-reported infertility and dietary recall data; residual confounding possible	Cited on the association between a dietary gut-microbiota index and female infertility
43	Salamun et al., 2022, Metab Syndr Relat Disord 20(7):384-394	Primary human observational study (RNA-sequencing/transcriptomic analysis)	Infertile women with obesity and PCOS (n=11) vs normal-weight controls (n=10); endometrial biopsies obtained during the window of implantation	Endometrial transcriptomic profiling of metabolic and inflammatory pathway genes	Endometrial transcriptome of obese women with PCOS showed dysregulation of metabolic and inflammatory pathway genes during the window of implantation compared with normal-weight controls	Small sample size; evidence specific to an obese PCOS population, not generalizable to obesity without PCOS; cross-sectional design	Supports the discussion in Section 3.4 of obesity/PCOS-associated endometrial transcriptomic dysregulation, with explicit caveat that this evidence derives from a PCOS population
44	Baik et al., 2023, Reprod Fertil 4(2):e210120	Human experimental/in vitro study (endometrial epithelial cell culture using microfluidics)	Human endometrial epithelial cells exposed to insulin in vitro (microfluidic culture system)	Insulin exposure and transcriptional response of endometrial epithelial cells	Insulin exposure altered the transcriptional response of endometrial epithelial cells, affecting pathways involved in protein export, glutathione metabolism, and ribosomal function	In vitro model; findings not yet validated in vivo; isolated insulin exposure does not replicate the full metabolic milieu of obesity	Supports the plausible mechanistic link, discussed in Section 3.4, between obesity-related hyperinsulinemia and endometrial gene expression, independently of PCOS status
45	Liu, Tan, He, 2024, Reprod Dev Med 8(3):162-168	Narrative review	General literature on endometrial macrophages and recurrent implantation failure (RIF); obesity not addressed	Macrophage polarization disorder in the endometrial immune microenvironment	Reviews evidence that disordered endometrial macrophage polarization toward a pro-inflammatory phenotype may contribute to recurrent implantation failure	Narrative review; not obesity-specific; evidence on endometrial macrophage polarization derives from other patient populations, not from women with obesity	Supports the cautious discussion in Section 3.4 of endometrial macrophage involvement in implantation, explicitly noting

Ref	Reference	Study design / evidence type	Population / experimental model	Exposure / intervention / topic	Key finding(s) relevant to this review	Main limitations	Relevance to the present review
							that direct evidence linking obesity to endometrial macrophage polarization remains indirect
46	Kanter et al., 2021, F S Rev 2(4):265-286	Narrative review	General literature on uterine natural killer (uNK) cell biology in early pregnancy; obesity not addressed	uNK cell biology, decidualization, angiogenesis, and early pregnancy establishment	Reviews the roles of uNK cells in decidualization, angiogenesis, and early trophoblast invasion during early pregnancy establishment	Narrative review; general uNK biology reference, not specific to obesity	Supports the general description of uNK cell function in Section 3.4, cited alongside ref. 29 (Perdu et al.), which specifically addresses obesity-related uNK alterations
